# Degree Correlations in Directed Scale-Free Networks

**DOI:** 10.1371/journal.pone.0110121

**Published:** 2014-10-13

**Authors:** Oliver Williams, Charo I. Del Genio

**Affiliations:** 1 Department of Physics, University of Warwick, Coventry, United Kingdom; 2 Warwick Mathematics Institute, University of Warwick, Coventry, United Kingdom; 3 Centre for Complexity Science, University of Warwick, Coventry, United Kingdom; 4 Warwick Infectious Disease Epidemiology Research (WIDER) Centre, University of Warwick, Coventry, United Kingdom; Universiteit Gent, Belgium

## Abstract

Scale-free networks, in which the distribution of the degrees obeys a power-law, are ubiquitous in the study of complex systems. One basic network property that relates to the structure of the links found is the degree assortativity, which is a measure of the correlation between the degrees of the nodes at the end of the links. Degree correlations are known to affect both the structure of a network and the dynamics of the processes supported thereon, including the resilience to damage, the spread of information and epidemics, and the efficiency of defence mechanisms. Nonetheless, while many studies focus on undirected scale-free networks, the interactions in real-world systems often have a directionality. Here, we investigate the dependence of the degree correlations on the power-law exponents in directed scale-free networks. To perform our study, we consider the problem of building directed networks with a prescribed degree distribution, providing a method for proper generation of power-law-distributed directed degree sequences. Applying this new method, we perform extensive numerical simulations, generating ensembles of directed scale-free networks with exponents between 2 and 3, and measuring ensemble averages of the Pearson correlation coefficients. Our results show that scale-free networks are on average uncorrelated across directed links for three of the four possible degree-degree correlations, namely in-degree to in-degree, in-degree to out-degree, and out-degree to out-degree. However, they exhibit anticorrelation between the number of outgoing connections and the number of incoming ones. The findings are consistent with an entropic origin for the observed disassortativity in biological and technological networks.

## Introduction

The use of networks is fundamental to model the structure and the dynamics of a vast number of systems found throughout the natural and engineered worlds. Their main appeal lies in allowing the reduction of a complex system to a discrete set of elements, the nodes, that interact across links. Then, one can study the structural properties of a network and infer results on the behaviour of the system thus modelled [1], [2]. The simplest global structural attribute of a network is its degree distribution *P*(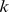), which expresses the probability of having a node with *k* links. A particularly important case is that of scale-free networks, in which the degree distribution obeys a power-law 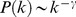 [3]–[6]. Scale-free networks have been observed in citation distributions [7]–[9], Internet and WWW topology [10], [11], biological systems [12], [13], technological, economic and social systems [14], [15], and transport processes [16], [17], and therefore they have been the subject of a considerable body of research. A generalization of the simple network model can be introduced by defining a directionality for the links. Directed networks are more suited to represent systems in which the interaction between elements is not necessarily symmetric, such as food webs or gene regulatory networks [18]. In this case, the connectivity of a node is no longer represented by a single scalar, as each node has a number of incoming connections (its in-degree 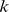 ^−^) and a number of outgoing connections (its out-degree 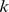 ^+^). A related quantity is the degree assortativity, often called simply assortativity, which measures the tendency of a node to be connected to nodes of similar degree. Assortativity is effectively a measure of the correlations amongst node degrees. As such, it is known to have substantial effects on the dynamical processes taking place on a network. For instance, assortative networks are more resistant to fragmentation in case of attack, while disassortative networks are less prone to cascading failures [19]–[21]. Degree correlations also play an important role in mathematical epidemiology, as they directly affect the dynamics of epidemic spreading, as well as the efficiency of defence mechanisms [22]–[25]. Numerous studies have shown that social networks are typically assortative, while biological and technological networks are disassortative, with links preferentially between nodes of high and low degree. In the case of directed networks, one can actually consider four different degree assortativities across links, as one can model the dependence of either in-degree or out-degree of a node on either in-degree or out-degree of its neighbours [26]–[28]. Here, we study how a scale-free structure affects assortativity in directed networks. In particular, we show that directed scale-free networks exhibit no in–in, out–out and in–out correlations, but are anticorrelated in the out–in assortativity.

## Methods

To study the preferred correlation structure induced by scale-freeness in directed networks, we performed extensive numerics, generating statistical ensembles of networks with power-law distributed in-degrees and out-degrees. The generation of directed networks with given degree distributions involves two distinct phases. First, extract two sequences of integer numbers that follow the distributions, and assign these to the nodes as directed half-links, or “stubs”. Taken in pairs, these numbers form a so-called bi-degree sequence, and correspond to the in-degree and the out-degree of each node. Then, sample the bi-degree sequence creating network realizations without self-edges or multiple edges. A suitable method to perform this second step is the algorithm discussed in Ref. [29], which allows an efficient uniform sampling of the realizations of a bi-degree sequence. However, not every sequence of integer pairs can be realized by a simple directed graph. Thus, before being able to apply the sampling algorithm, we need to develop a procedure to properly create bi-degree sequences that admit realizations, which are referred to as graphical. To do so, we start from the Fulkerson theorem [30], which states the necessary and sufficient graphicality conditions for bi-degree sequences:

### Theorem 1

*A sequence of non-negative integer pairs* 
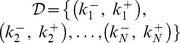
 *with* 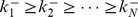 *is graphical if and only if*

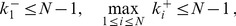
(1)

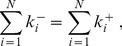
(2)

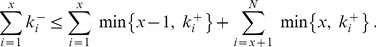
(3)


Theorem 1 can be used to efficiently verify the graphicality of an extracted sequence using the particularly fast implementation described in [29]. The theorem can be intuitively understood by looking at the three conditions. Condition 1 simply ensures that no node has a number of incoming or outgoing stubs that exceeds the number of other nodes. This is clearly a necessary condition, since each node can connect to or receive connections from at most all the remaining nodes. To understand Condition 3, notice that the left-hand side is just the number of incoming stubs in the set consisting of the first *x* nodes. Then, consider how to maximize this number. To start with, take each of the first *x* nodes and connect them to all the others in the set. However, note that each node *i* can only have as many outgoing connections as its out-degree 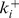. Thus, if the out-degree of node *i* is large enough, it can be connected to all the remaining *x*−1 nodes; otherwise, it can only be connected to 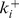 amongst the remaining *x*−1. The first term in the sum on the right-hand side accounts for these connections. The second term in the sum has the same meaning. However, the sum is now taken over the nodes that are *not* within the first *x*. Thus, each can connect to at most *x*, rather than *x*−1, other nodes. Finally, Condition 2 mandates the total number of incoming stubs equal the total number of outgoing stubs. This introduces an important constraint in the generation of graphical bi-degree sequences. To see why, write the number of incoming and outgoing stubs as
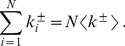
(4)


In general, the power-law exponents for in-degrees and out-degrees in directed scale-free networks can be different [1]–[5]. Then, if the out-degrees scale as 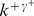 and the in-degrees as 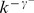, for 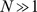, it is 
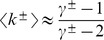
. Thus, one cannot expect the sums of in-degrees and out-degrees to be equal, if the respective power-law exponents are different. In fact, in the region of interest 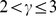, the variance of power-law distributions is unbounded, since 
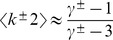
. Thus, in this range of exponents, one should not expect Condition 2 to be satisfied even when choosing the same exponent for in-degrees and out-degrees.

To guarantee that Condition 2 is satisfied, and avoid the trivial non-graphicality of the generated bi-degree sequence, one cannot extract independently the sequences of in-degrees and out-degrees. Rather, one should be extracted without further constraints, and the other should be conditioned to have the same sum as the former. Without loss of generality, assume that 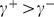. Then, it is 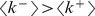. Thus, freely extracting the out-degrees requires, on average, to lower the mean in-degree with respect to its unconstrained value. This effectively introduces an upper cutoff excluding all the degrees above a certain threshold 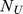. For a large network, the normalization constant of the in-degree distribution with an upper cutoff is
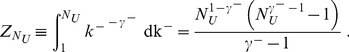
(5)


Thus, the mean in-degree is
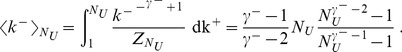
(6)


Equating Eq. 6 with the expression for the unconstrained mean out-degree yields
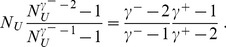
(7)


**Figure 1:**
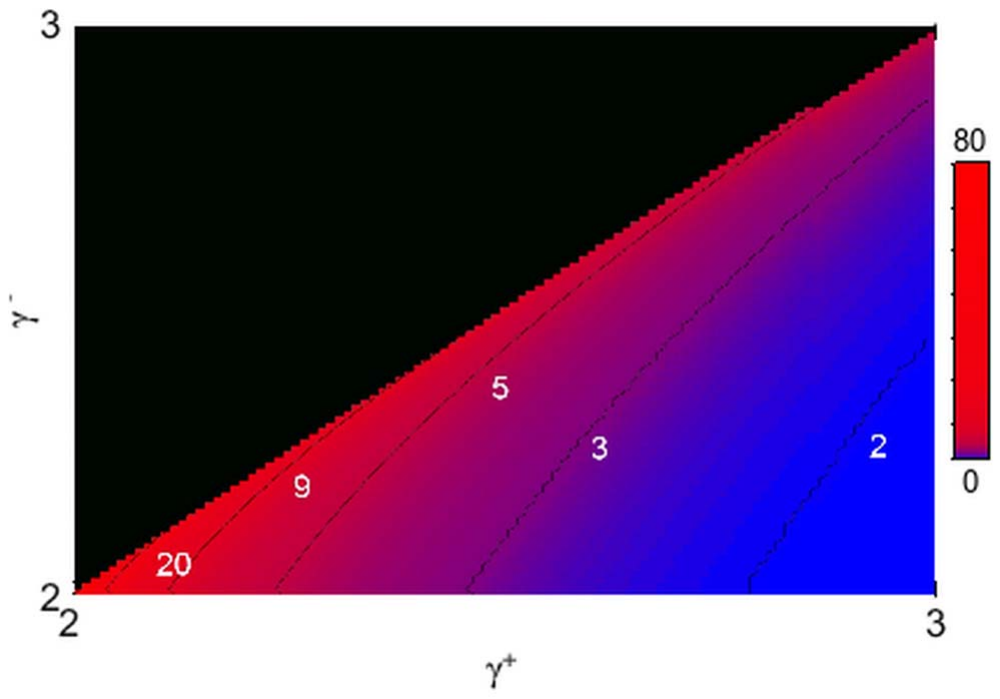
Effective upper cutoff on the in-degrees if they are conditioned on the sum of the out-degrees. The contour plot shows the logarithm of the introduced upper cutoff. Note that for almost all the choices of power-law exponents, such cutoff is so low that the greatest part of the distribution tail is lost, affecting the scale-free character of the resulting network. The labels indicate the logarithm of the cutoff for the corresponding contour lines. Only half of the region is plotted, as we are under the assumption that 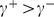.

The solution to Eq. 7, plotted in Fig. 1, show that for almost all the choices of 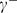 and 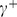, the upper cutoff would eliminate the vast majority of the tail of the degree distribution. As the defining characteristic of scale-free networks is a power-law tail, this indicates that the choice of conditioning the in-degree distribution on the sum of the out-degrees is not suitable for sequence generation.

The other possibility is extracting the in-degrees in an unconstrained way, and conditioning the out-degrees on their sum. This time, the cutoff introduced is a lower cutoff 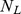 on the out-degree distribution. For 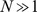, the out-degree normalization constant with lower cutoff is
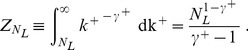
(8)


Then, the mean out-degree is
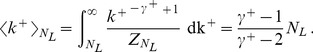
(9)


As the two mean degrees have to be equal, it is
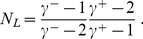
(10)


Note that, defining the excess exponent 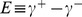, the equation above can be rewritten as
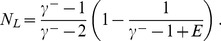
(11)


**Figure 2:**
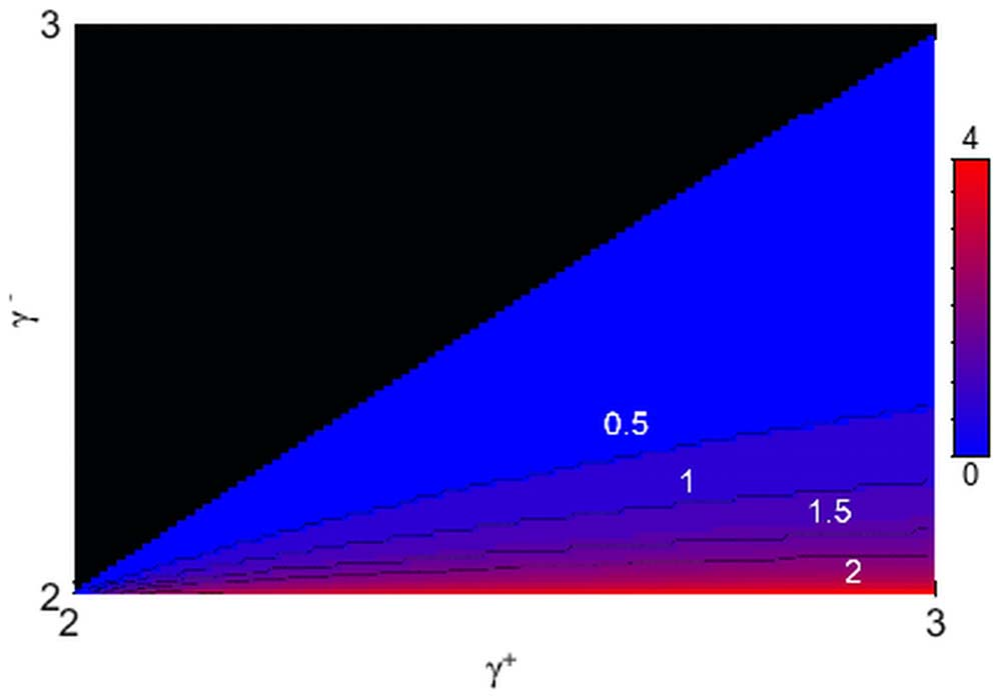
Effective lower cutoff on the out-degrees if they are conditioned on the sum of the in-degrees. The contour plot shows the logarithm of the introduced lower cutoff. Unlike what happens with the reverse choice, the cutoff introduced is always minor, and it actually vanishes for most of the choices of in-degree and out-degree exponents. The labels indicate the logarithm of the cutoff for the corresponding contour lines. Only half of the region is plotted, as we are under the assumption that 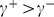.

This form explicitly shows that *N_L_* is 1 when the exponents are equal and *E* = 0, and it increases monotonically with *E*, towards an asymptotic value of 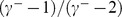. As illustrated in Fig. 2, such cutoff is very mild. Thus, this approach leaves the tail of the distribution entirely untouched. Moreover, for more than half of the region of interest, the whole out-degree distribution has no effective cutoff at all.

Notice that defining a proper method for the generation of power-law distributed directed degree sequences is essential for the accuracy of research outcomes. In fact, approximate techniques have uncontrolled errors and produce results that depend on the details of the approximation made [18], [31].

## Results and Discussion

At the light of the considerations expressed in the previous section, we generated ensembles of bi-degree sequences of random power-law distributed integers with exponents between 2 and 3, conditioning the sequence with the greater exponent on the sum of the sequence with the lower one. Then we tested the sequences for graphicality, and sampled the graphical ones using the direct construction algorithm detailed in Ref. [29]. For each sample, we measured the assortativities using the Pearson coefficients
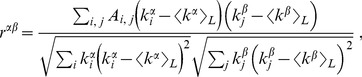
(12)where the averages are taken over all directed links, the elements of the adjacency matrix of the network *A_i,j_* are 1 if there is a link from node *i* to node *j*, and *α* and *β* can be − or +, indicating in-degrees or out-degrees, respectively [32]. We stress that the sampling method used is a degree-based graph construction algorithm [33]. Algorithms in this class can access the entire space of the realizations of a graphical sequence. They work by building the sample graphs via the systematic placement of links, guaranteeing that the graphicality of the sequence is maintained after each step. At every moment, the combinatorially exact probabilities of placing each allowed link are completely determined. Thus, these methods efficiently allow uniform graph sampling, without introducing biases due to a particular choice of generative model or construction algorithm, which can result in overrepresentation or inaccessibility of part of the realization space.

**Figure 3:**
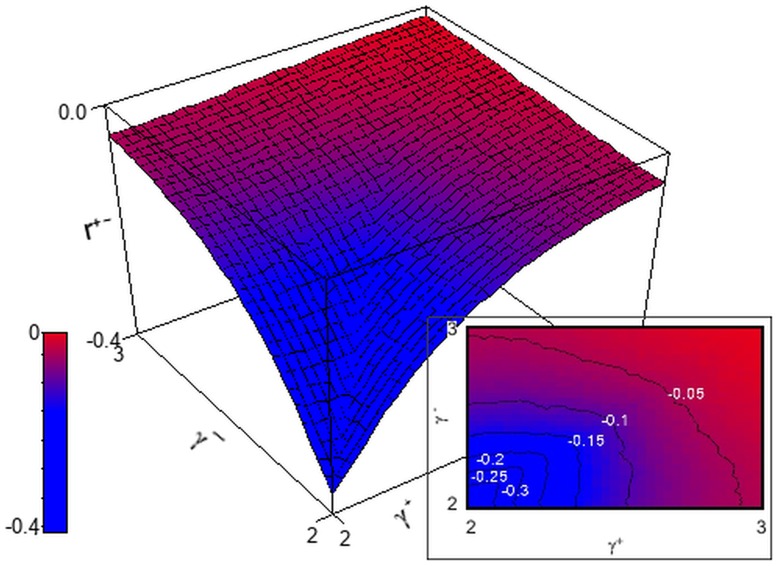
Degree correlations in directed scale-free networks. The Pearson correlation coefficient *r* ^+−^ is always negative, indicating that directed scale-free networks are naturally disassortative when one considers the out-in correlation. The inset shows a contour plot of the same data, for added clarity. The labels in the contour plot indicate the value of *r* ^+−^ for the corresponding contour lines.

**Figure 4:**
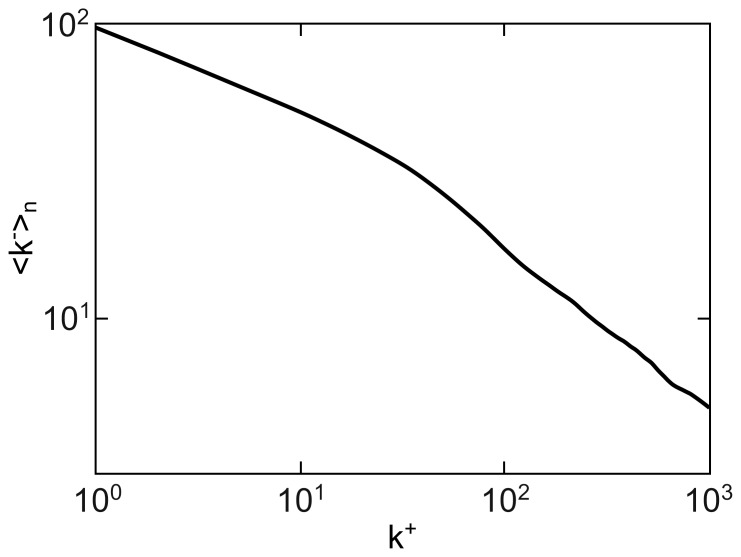
Disassortative degree correlations. The plot shows the average in-degree 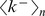 of the neighbours of nodes with a given out-degree 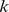 ^+^ for an ensemble of networks with 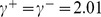 and *N* = 1000. The dependence of 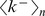 on 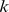 ^+^ clearly indicates that nodes with low out-degree link preferentially to nodes of high in-degree, and nodes with high out-degree link mostly to nodes of low in-degree. The monotonically decreasing dependence confirms the strong disassortative nature of the networks.

The results indicate the absence of any dependence of the in-in, in-out and out-out coefficients on the choice of power-law exponents. In fact, these three coefficients all vanish within the uncertainties throughout the region studied. Conversely, the out-in coefficient is always negative (Fig. 3), indicating disassortative correlation between the out-degree of the node at the beginning of a link and the in-degree of the node at its end. Figure 4 illustrates this pattern of dependence by plotting the average in-degree 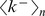 of the neighbours of nodes with a given out-degree 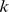 ^+^, for an ensemble of networks with 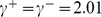 and *N* = 1000. The values of 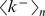 decrease quickly and monotonically with 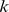 ^+^, confirming the strong disassortative nature of the networks. Our results show substantial similarities between the correlation structure of directed and undirected scale-free networks. Indeed, it is a well-known fact that random undirected scale-free networks are disassortative [34]–[38]. Thus, to explain our findings, we use the entropic treatment described in Ref. [39], extending it to directed networks. To do so, write the information entropy of a given network as
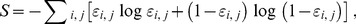
(13)where 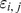 is the expectation value for the 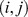 element of the adjacency matrix. To derive an expression for 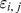 in the case of a given bi-degree sequence, note that it has to satisfy two conditions, namely
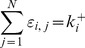
(14)and
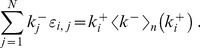
(15)


A form that satisfies these conditions is
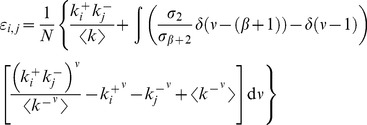
(16)where 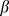 is a free parameter. In principle, the factor 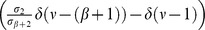 in Eq. 16 can be replaced by any arbitrary function of 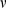. However, the choice made, in which 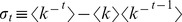, allows to reproduce the observed dependence of 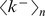 on 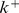. Then, computing the integral in Eq. 16, it is
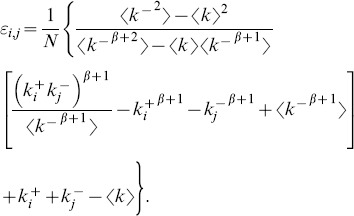
(17)


**Figure 5:**
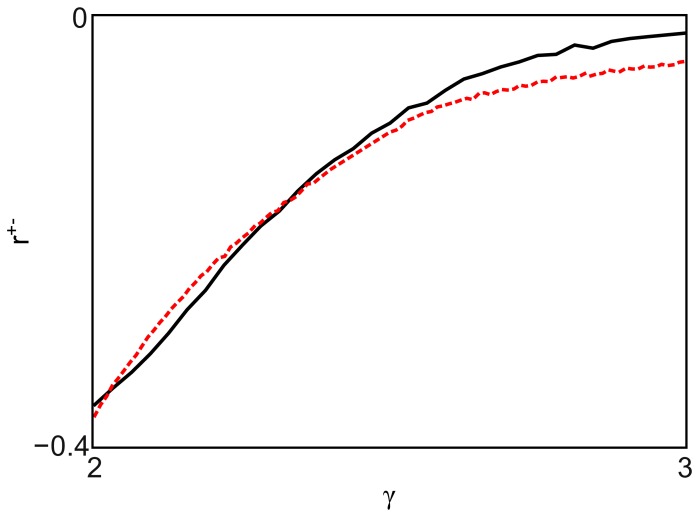
Entropy-maximizing disassortativity. The plot shows the out-in correlation coefficients for directed scale-free networks with 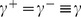. The simulation data are shown in solid black. The red dotted line corresponds to the coefficients that maximize the information entropy for a given 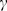. The good agreement of the results indicates the entropic origin of the disassortativity observed in directed scale-free networks.

Using Eqs. 13 and 17, we can find the choice of *β* that maximizes *S*, and compute the Pearson coefficient *r* ^*^ corresponding to the maximum entropy network for any given power-law exponents. Notice that in the equations above, we make no distinction between 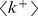 and 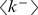, as they must be equal to ensure graphicality of the bi-degree sequence. Also, we restrict the parameter search to the values that yield networks without self-edges or multiple edges. To carry out the calculation, we use the degree-maximizing sequence as representative of the scale-free networks for each value of 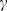 [6]. Figure 5 displays a comparison between 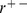 as measured by simulations and *r* ^*^, in the case of 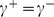. The two sets of results are substantially in agreement, save for higher values of 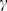, where 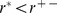. This can be explained by considering that the degree-maximizing sequences used to compute *r* ^*^ feature more high-degree nodes than would be found on average, thus decreasing the assortativity of their realizations.

In summary, we showed that directed scale-free networks are naturally uncorrelated when considering in-in, in-out and out-out correlations. Thus, when looking across a directed link, the in-degree of the originating node has no influence on the in-degree or the out-degree of the target node. Similarly, the out-degrees are not affected by the out-degrees of the neighbours. However, the out-in correlation coefficient is found to be negative throughout the region studied. This indicates that the natural state of directed scale-free networks is one in which nodes of low degree prefer to link to nodes of high degree, and vice versa. The origin of this preference is entropic, as the coefficients found are in good agreement with those corresponding to the maximum information entropy. Thus, the observation of a disassortative directed scale-free network is not sufficient to infer the existence of extra growth mechanisms beyond those responsible for its degree distribution. These results suggest that the disassortative correlations observed in many real-world systems, such as biological and technological networks, do not necessarily arise because of design or evolutionary pressure. In fact, the absence of such drivers, and the resulting randomness, would lead to the observation of the anticorrelated state as the most probable one. Notice that this does not exclude the presence of evolutionary mechanisms, which may certainly be the cause of an observed disassortative network topology in some specific cases. However, their action would have to promote the maximum-entropy state, thus making their presence undetectable from the degree distribution and correlations alone.

## Supporting Information

File S1 Raw data files.
(GZ) Click here for additional data file.
